# Impacts of an Exercise and Behavioral Intervention on Community‐Dwelling Older Adults with Dementia and Their Family Caregivers in Thailand

**DOI:** 10.1002/alz.085980

**Published:** 2025-01-09

**Authors:** Hongtu Chen, Sue E Levkoff, Siranee Sihapark, Ladson Hinton, Sirinart Thongsiri, Bussabong Wisetpholchai, Linda Teri, Dolores Gallagher‐Thompson, Suthida Intaraphet, Kaipeng Wang, Komatra Chuengsatiansup

**Affiliations:** ^1^ Harvard Medical School, Boston, MA USA; ^2^ University of South Carolina, Columbia, SC USA; ^3^ Boromarajonani College, Khon Kaen Thailand; ^4^ University of California, Davis, Sacramento, CA USA; ^5^ Mahasarakham University, Maha Sarakham Thailand; ^6^ Thai Health Academy, Bangkok Thailand; ^7^ University of Washington, Seattle, WA USA; ^8^ University of California at Davis Betty Irene Moore School of Nursing, Davis, CA USA; ^9^ University of Denver, Denver, CO USA; ^10^ Sirindhorn Anthropology Center, Bangkok Thailand

## Abstract

**Background:**

This study uses the data collected from the “*P*artnership in *I*mplementation *S*cience for *G*eriatric *M*ental *H*ealth (PRISM)” project, a randomized trial designed to test implementation support strategies for the delivery of the Reducing Disability in Alzheimer’s Disease (RDAD) program, an evidence‐based multi‐component exercise and behavioral/psychosocial intervention for older adults with dementia and their family caregivers in Thailand.

**Method:**

A total of 353 dyads of persons with dementia (PwD) and behavioral and psychological symptoms of dementia (BPSD) and their family caregivers received a 12‐week RDAD intervention and were assessed at baseline, and at 3‐ and 6‐months post‐treatment. Longitudinal analyses were conducted using paired‐sample t‐tests to estimate the changes in each of the outcomes by treatment groups.

**Result:**

Paired t‐tests showed that between baseline and 3 months, there were significant decreases in caregiver clinical outcomes, including caregiver burden (p = 0.047), depressive symptoms (p = 0.02), and BPSD‐related caregiver distress (p < 0.001). Similarly, there were significant decreases between baseline and 3 months in PwD’s mental health indicators, including BPSD symptoms (p < 0.001), BPSD severity (p < 0.002), and depression (p < 0.001). PwDs also showed a significant increase in physical function assessed by ADLs (p < 0.031) and cognitive function, as assessed by the Thai MMSE (TMSE) (p < 0.001), and these trends persisted between the 3‐ and 6‐month follow‐up assessments, with significant increases in ADLs (p < 0.001) and TMSE (p < 0.001). We also conducted heterogeneity regression analysis to assess whether the time changes might be determined by baseline characteristics (e.g., sex, age, subgroups) and found no significant influences of these factors on changes in outcomes over time.

**Conclusion:**

The results suggest that the RDAD intervention significantly reduces caregivers’ psychological burden, depressive symptoms, and BPSD‐related distress and also improves the PwD’s mental, physical, and cognitive function. These findings provide evidence that supports efforts to expand the reach of US‐developed evidence‐based non‐pharmacological interventions to improve dementia care for community dwellers in resource‐limited settings like Thailand.

